# Synergistic Effect of Lithocholic Acid with Gentamicin against Gram-Positive Bacteria but Not against Gram-Negative Bacteria

**DOI:** 10.3390/molecules27072318

**Published:** 2022-04-03

**Authors:** Hongfa Lv, Lianping Wang, Shuang Liu, Wei Hu, Jianfeng Wang, Xuming Deng, Jinying Gao

**Affiliations:** 1 Department of Respiratory Medicine, The First Hospital of Jilin University, Changchun 130021, China; lvhf139@163.com (H.L.); 15661134131@163.com (W.H.); wjf927@jlu.edu.cn (J.W.); dengxm@jlu.edu.cn (X.D.); 2 Jilin Ginseng Academy, Chang Chun University of Chinese Medicine, Changchun 130117, China; ziliaowlp@163.com; 3 Jilin Jinziyuan Biotech Inc, Changchun 136400, China; durb1995@163.com

**Keywords:** lithocholic acid, *Listeria monocytogenes*, antibiofilm, gentamycin adjuvant, food-borne disease

## Abstract

*Listeria monocytogenes* (*L. monocytogenes*) is an important Gram-positive food-borne pathogen that severely threatens public health. A checkerboard microdilution method was performed to evaluate the synergistic effect of lithocholic acid (LCA) with Gentamicin (Genta) against *L. monocytogenes*. BacLight LIVE/DEAD staining, scanning electron microscopy and biofilm inhibition assays were further used to explore the bactericidal effect and antibiofilm effect of this combination on *L. monocytogenes*. Additionally, the synergistic effects of LCA derivatives with Genta were also evaluated against *L. monocytogenes*, *S.* *aureus* and *S. suis.* The results indicated that a synergistic bactericidal effect was observed for the combined therapy of LCA at the concentration without affecting bacteria viability, with Genta. Additionally, LCA in combination with Genta had a synergistic effect against Gram-positive bacteria (*L. monocytogenes*, *S. aureus* and *S. suis*) but not against Gram-negative bacteria (*E. coli*, *A. baumannii* and *Salmonella*). BacLight LIVE/DEAD staining and scanning electron microscopy analysis revealed that the combination of LCA with Genta caused *L. monocytogenes* membrane injury, leading to bacteria death. We found that 8 μg/mL LCA treatment effectively improved the ability of Genta to eradicate *L. monocytogenes* biofilms. In addition, we found that chenodeoxycholic acid, as a cholic acid derivative, also improved the bactericidal effect of Genta against Gram-positive bacteria. Our results indicate that LCA represents a broad-spectrum adjuvant with Genta for infection caused by *L. monocytogenes* and other Gram-positive pathogens.

## 1. Introduction

Food-borne diseases caused by microorganisms or chemicals have been recognized as a grave issue for human health due to the intake of contaminated food stuffs. Increasing numbers of people are suffering from food-borne diseases, especially in undeveloped countries [1], thus, resulting in considerable economic losses and decreased productivity [2]. Ingestion of food contaminated by *Listeria monocytogenes*, *Salmonella* or *Escherichia coli* can lead to food-borne infections with manifestations such as sickness, dizziness, stomachache and diarrhea [3]. Thus, the development of agents or strategies for treating infections by these food-borne bacteria is urgent.

*L. monocytogenes*, the agent of listeriosis, is a severe food-borne pathogen and can cause digestive-system problems. *L. monocytogenes* is a chief cause of abortion, meningitis and neonatal death among immuno-compromised patients and pregnant women [4]. Listeriosis generally shows unobvious symptoms in pregnant woman, however, with fetal lethality as high as 35% [4]. Additionally, the biofilm formation of *L. monocytogenes* is possible in surfaces of stainless steel, polyethylene and pipelines in food-processing facilities. Biofilm formation renders this bacterium resistant to antimicrobials and the host immune system, which further contributes to the survival and reproduction of the bacterial cells in the host [5]. Due to the resistance to various hostile environments, *L. monocytogenes* biofilms are a daunting food safety issue to be addressed.

Measures are necessary to prevent and disturb *L. monocytogenes* biofilm formation. Small molecule compounds originating from natural sources have been reported to possess the capability of promoting bacterial detachment from the substratum and interfering with biofilm maturity by inhibiting genes expression in the quorum sensing system [3]. Topical antibiotic therapy was used for biofilm-associated bacterial infection with increasing doses [6]. However, such a strategy is not effective for infection located in deep tissues or avascular areas where it is difficult for antibiotics to reach. Significantly, the combination of antibacterial synergists with antibiotics against bacteria biofilm formation could be an effective strategy [7].

Bile acids are formed from cholesterol in the mammalian liver by neutral and acidic pathways [8]. Then, cholic acid and chenodeoxycholic acid, as a primary bile acids, combined with glycine or taurine, are secreted into the intestine. Following metabolism by the gut microbiota, deoxycholic acid and LCA are synthesized [9]. Due to their good biocompatibility, bile acids derivatives with large steroid backbones and facial amphiphilic structure can penetrate into bacterial membranes and exert antibacterial effects [10].

Cholic acid derivatives isolated from *Bacillus amyloliquefaciens* cultures showed antimicrobial effect against *P. aeruginosa* and *B. cereus* [11]. LCA, as the secondary metabolite of bile acids, has been reported to possess antibacterial activity against *S. aureus* in combination with amikacin [12]. In addition, LCA has been shown to have anti-inflammatory [13], intestinal phosphate and calcium absorption improvement [14] and antiviral effects [15]. In our study, we explore multiple combinations of LCA with various antibiotics for both Gram-positive bacteria and Gram-negative bacteria. Furthermore, the synergistic bactericidal and antibiofilm effects between LCA and Genta against *L. monocytogenes* are determined, to aid in ushering the development anti-infectious agents against foodborne pathogens.

## 2. Results

### 2.1. LCA Improves L. monocytogenes Sensitivity to Genta In Vitro

**Figure 1:**
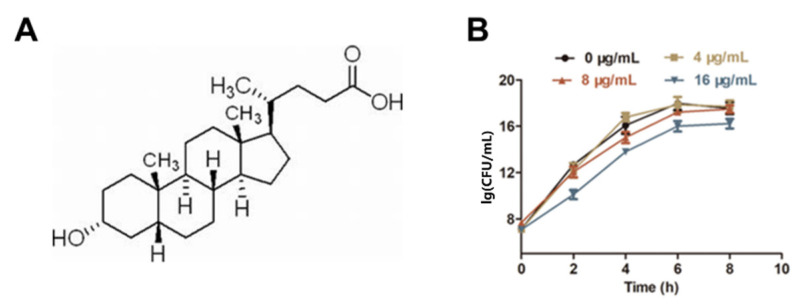
LCA had no influence on the growth of *L. monocytogenes* at a concentration of less than 16 µg/mL. (**A**) Chemical structure of LCA. (**B**) Growth curve of *L. monocytogenes* cultured in TSB containing the indicated concentrations of LCA.

**Figure 2:**
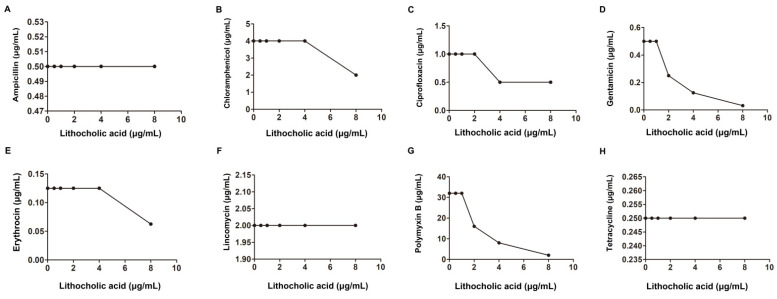
Interaction of LCA with Amp, Ery, Genta, Cip, Lin, Tet, Cpl and Pmb against *L. monocytogenes* (**A**–**H**).

The results of a checkerboard microdilution assay indicated that LCA (Figure 1A), at a concentration of 8 μg/mL, was identified as an effective synergistic inhibitor with Genta against *L. monocytogenes* (FIC index = 0.5) (Figure 2D and Table 1). However, the MIC of LCA against *L. monocytogenes* was 32 μg/mL, and no visible inhibition of *L. monocytogenes* growth was observed at 8 μg/mL from the results of the growth curve assay (Figure 1B).

Thus, LCA, at a concentration without affecting bacterial viability, combined with Genta, had a synergistic antibacterial effect on *L. monocytogenes*. Additionally, the potential synergistic antibacterial effect of LCA with other antibiotics against *L. monocytogenes* was further determined. As shown in Figure 2 and Table 1, in agreement with the Genta results, LCA combined with Pmb (Polymyxin B) showed a synergistic bactericidal effect against *L. monocytogenes* with an FIC index of 0.5. However, such synergistic effects were not observed for other antibiotics, including Amp (Ampicillin), Ery (Erythromycin), Cip (Ciprofloxacin), Lin (Lincomycin), Tet (Tetracycline) and Cpl (Chloramphenicol).

**Figure 3:**
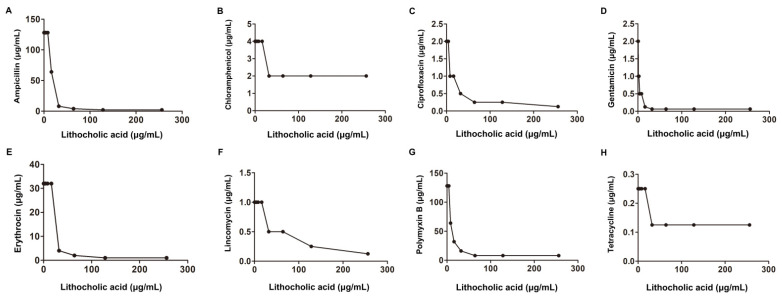
Interaction of LCA with Amp, Ery, Genta, Cip, Lin, Tet, Cpl and Pmb against *S. aureus* (**A**–**H**).

**Figure 4:**
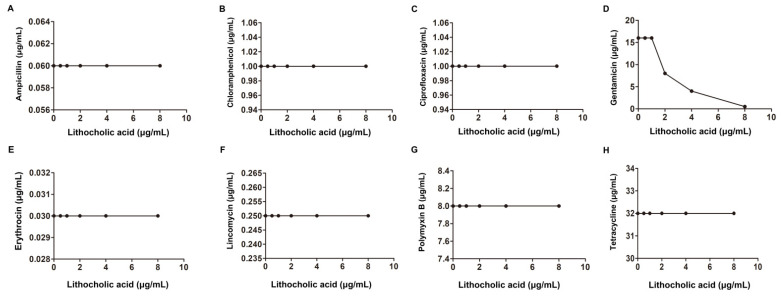
Interaction of LCA with Amp, Ery, Genta, Cip, Lin, Tet, Cpl and Pmb against *S. suis* (**A**–**H**).

Another checkerboard microdilution analysis was conducted to further explore whether a synergistic effect also occurred in the other bacteria with the above tested antibiotics in combination with LCA. As shown in Figure 3, Table 1 and Table 2, LCA combined with Amp, Cip, Genta, Ery or Pmb had synergistic bactericidal effects against *S. aureus* (0.15 < FIC index < 0.37) but not when combined with Lin, Tet or Cpl. For another Gram-positive bacterium, *S. suis*, a synergistic bactericidal effect was only observed with Genta with 4 μg/mL LCA with an FIC index of 0.5 (Figure 4 and Table 1). 

**Figure 5:**
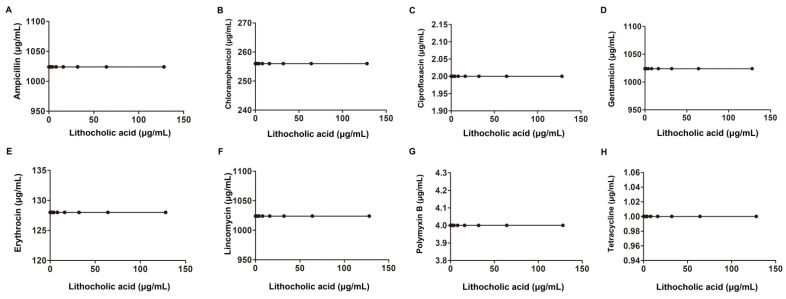
Interaction of LCA with Amp, Ery, Genta, Cip, Lin, Tet, Cpl and Pmb against *E. coli* ATCC25922 (**A**–**H**).

**Figure 6:**
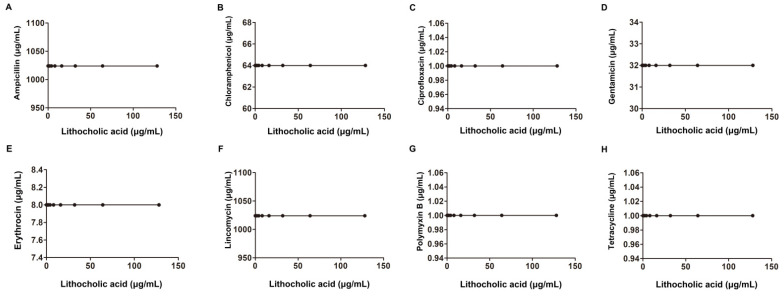
Interaction of LCA with Amp, Ery, Genta, Cip, Lin, Tet, Cpl and Pmb against *Acinetobacter baumannii* ATCC19606 (**A**–**H**).

**Figure 7:**
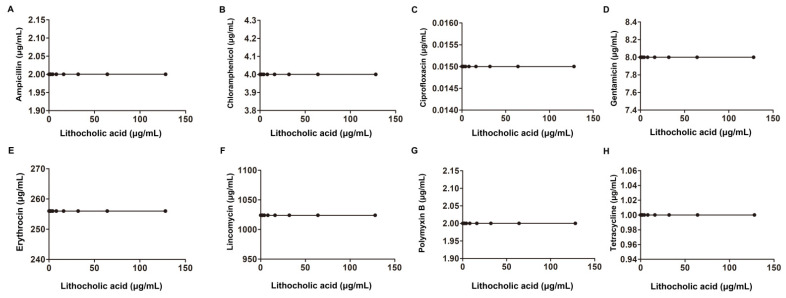
Interaction of LCA with Amp, Ery, Genta, Cip, Lin, Tet, Cpl and Pmb against *Salmonella* SL1344 (**A**–**H**).

Gram-negative bacteria, including *E. coli*, *A. baumannii* and *Salmonella,* were also examined, and no synergistic bactericidal effect was observed for any of these tested Gram-negative bacteria (Figure 5, Figure 6 and Figure 7 and Table 2). Taken together, our results found that LCA combined with Genta had a synergistic bactericidal effect on *L. monocytogenes*, *S. aureus* and *S. suis*, suggesting that LCA treatment could improve the sensitivity of Gram-positive bacteria to Genta.

### 2.2. LCA in Combination with Genta Cause Cell Membrane Injure of L. monocytogenes

**Figure 8:**
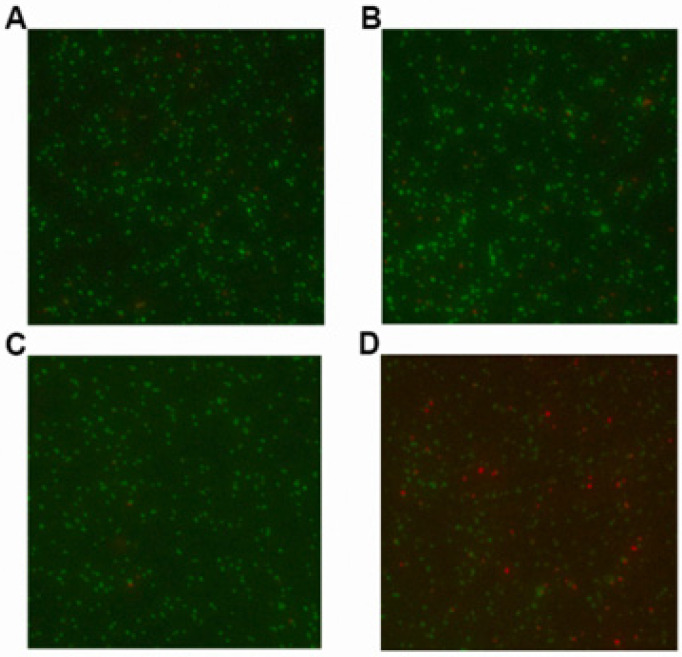
Synergistic bactericidal effects of LCA combined with Genta against *L. monocytogenes*. The live/dead bacteria with the following treatment were stained with a BacLight LIVE/DEAD staining kit. (**A**) The untreated control. (**B**) *L. monocytogenes* treated with 8 μg/mL LCA at 37 °C for 3 h. (**C**) *L. monocytogenes* treated with 0.125 μg/mL Genta at 37 °C for 3 h. (**D**) *L. monocytogenes* treated with 8 μg/mL LCA and 0.125 μg/mL Genta at 37 °C for 3 h.

BacLight LIVE/DEAD staining experiments were conducted to visualize the cell membrane injure of LCA combined with Genta against *L. monocytogenes*. In principle, bacteria with intact cell membranes fluoresce bright green, whereas dead cells with compromised membranes fluoresce red. Consistent with the antibacterial activity analysis, *L. monocytogenes* treated with 8 μg/mL LCA were dyed green (live) (Figure 8B), which was similar to the sample without any treatment (Figure 8A). 

In contrast to Figure 8A, a few bacteria were injured or dead, as evidenced by red fluorescence (dead), in the sample treated with Genta at a concentration of 0.125 μg/mL, which was less than the MIC of Genta against *L. monocytogenes* (0.5 μg/mL) (Figure 8C). As expected, there were many more bacteria dyed red (dead) in the samples treated with 8 μg/mL LCA and 0.125 μg/mL Genta (Figure 8D) compared to Figure 8B,C, suggesting that LCA combined with Genta had a synergistic bactericidal effect on *L. monocytogenes* and caused cell membrane injury of *L. monocytogenes*.

### 2.3. LCA Combined with Genta Induces Morphological Changes in L. monocytogenes

**Figure 9:**
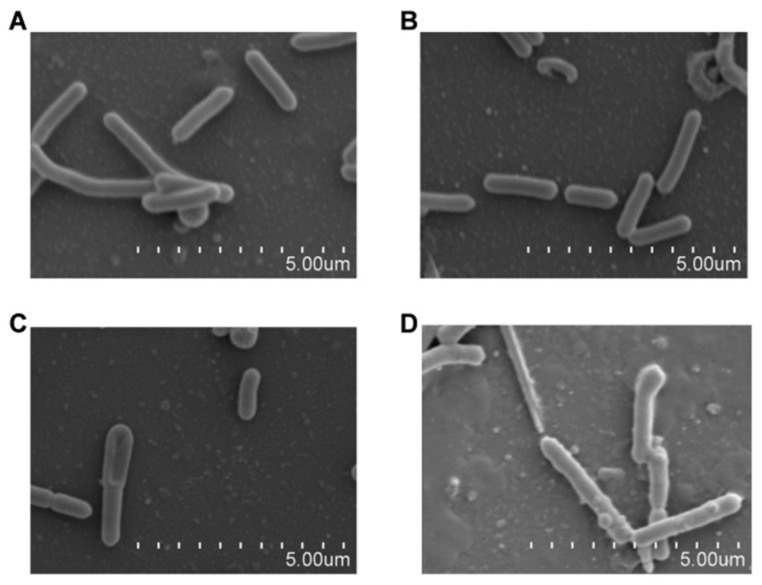
LCA combined with Genta induced *L. monocytogenes* membrane injury as observed by scanning electron microscopy. (**A**) Normal morphology of *L. monocytogenes*. (**B**) Surface image of *L. monocytogenes* after treatment with 8 μg/mL LCA. (**C**) Surface image of *L. monocytogenes* after treatment with 0.125 μg/mL Genta. (**D**) Rupture of the membrane of *L. monocytogenes* following treatment with 8 μg/mL LCA and 0.125 μg/mL Genta.

The morphology of *L. monocytogenes* was further observed under scanning electron microscopy to examine the potential bactericidal effect of LCA in combination with Genta. As shown in Figure 9A, the untreated *L. monocytogenes* was well circumscribed with smooth membrane surfaces without swelling. In samples treated with 8 μg/mL LCA, the morphology of *L. monocytogenes* was similar to that of bacteria without treatment (Figure 9B). In addition, an injured membrane was observed in *L. monocytogenes* treated with 0.125 μg/mL Genta with deformed morphology and slight swelling, which are signs indicative of cell death (Figure 9C). 

Consistent with the above results, as shown in Figure 9D, LCA combined with Genta led to significant cell death without intact bacterial morphology compared to bacteria treated with LCA or Genta alone. Taken together, our results establish that LCA combined with Genta demonstrated synergistic bactericidal effects that resulted in injury to the bacterial membrane leading to bacterial death.

### 2.4. LCA Improves the Inhibition of L. monocytogenes Biofilm Formation by Genta

**Figure 10:**
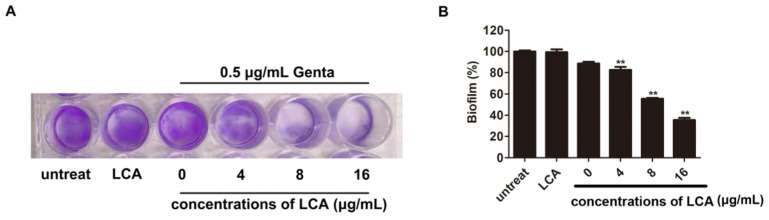
LCA combined with Genta inhibited *L. monocytogenes* biofilm formation. (**A**) Overall image of *L. monocytogenes* biofilms with the indicated treatment. (**B**) *L. monocytogenes* biofilms were quantified by determining the absorbance value of crystal violet at 570 nm, **p < 0.01.

The formation of biofilms is critical for *L. monocytogenes* defense against hostile in vitro situations and the *in vivo* host immune system [16]. Thus, whether *L. monocytogenes* biofilms are inhibited by the combination of LCA and Genta was further determined. *L. monocytogenes* biofilms were observed after incubation in 24-well plates. However, 0.5 μg/mL Genta or 16 μg/mL LCA did not disturb the biofilm formation of *L. monocytogenes*, as demonstrated by the lack of statistical significance for the intensity of crystal violet compared to the control without treatment. Under 0.5 μg/mL Genta treatment, 4 μg/mL LCA had no significant effect on *L. monocytogenes* biofilm formation (Figure 10A). 

Interestingly, the combined treatment of 0.5 μg/mL Genta with 8 μg/mL LCA or 16 μg/mL LCA visibly inhibited *L. monocytogenes* biofilm formation, as shown in Figure 10A. Biofilm biomass was further quantified by determining the absorbance value of crystal violet in each sample. In agreement with the gross observation, 8 μg/mL LCA or 16 μg/mL LCA in combination with Genta treatment significantly decreased the absorbance value of crystal violet at 570 nm from 2.8 to 1.5 or 0.9 (*p* < 0.05), respectively, compared to untreated bacteria (Figure 10B). Thus, LCA combined with Genta significantly inhibited *L. monocytogenes* biofilm formation.

### 2.5. Chenodeoxycholic Acid Also Improves the Bactericidal Effect of Genta against Gram-Positive Bacteria

**Figure 11:**
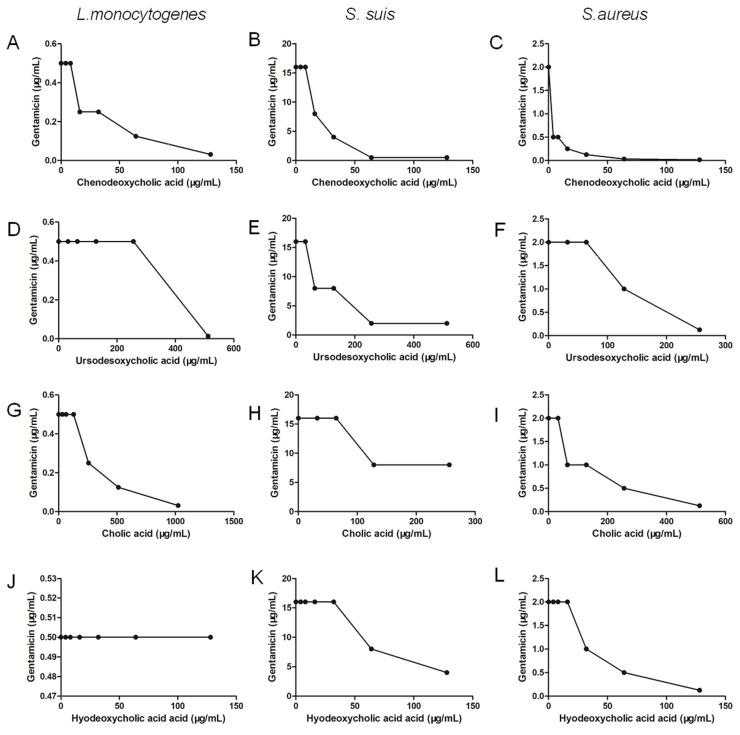
Interaction of LCA derivatives and Genta against *L. monocytogenes* (**A**,**D**,**G**,**J**), *S. suis* (**B**,**E**,**H**,**K**) and *S. aureus* (**C**,**F**,**I**,**L**).

From our results in Figure 11 and Table 3, we found that chenodeoxycholic acid as a cholic acid derivative also improved the bactericidal effect of Genta against Gram-positive bacteria, including *L. monocytogenes*, *S. aureus* and *S. suis*, whose FIC index values were 0.5, 0.26 and 0.28, respectively, in Table 3. Hyodeoxycholic acid also improved the bactericidal effect of Genta against *S. aureus* (FIC index = 0.5) but not on *L. monocytogenes* and *S. suis* in Table 3. However, ursodeoxycholic acid and cholic acid had no synergistic effect against the tested strains in Table 3.

## 3. Discussion

Research has found that 66% of foodborne disease is caused by bacteria [17]. Listeriosis as a foodborne disease. The cost for the treatment of listeriosis reached 22 billion dollars per year, which was slightly lower than that of *Clostridium botulinum* and *Vibrio vulnificus* treatment in the USA [18,19]. Biofilm formation further facilitates that ability of *L. monocytogenes* to overcome harsh environments and the host immune system. Thus, *L. monocytogenes*, a main foodborne pathogen, poses a great challenge for the food processing industry and public health.

To address biofilm infections caused by *L. monocytogenes*, our study determined that LCA combined with Genta effectively exerted bactericidal effects and inhibited biofilm formation at a concentration of 8 μg/mL. The biofilm as a “house” protected bacteria from antibiotic, sanitizer and desiccation as well as other adverse factors. However, no antibiotic targeted to biofilm infections was prepared [20]. Antimicrobial peptides, surfactants (SDS, Tween 20 and Triton X-100), free fatty acids and amino acids were utilized to inhibit biofilm formation [20]. However, the antibiofilm combination strategy to inhibit biofilm formation is an effective method compared with when they are used alone [21]. Therefore, LCA combined with Genta could be a new method to combat *L. monocytogenes* biofilm infections.

In terms of other foodborne pathogens, LCA had a synergistic bactericidal effect on *S. aureus* when combined with several antibiotics, including Amp, Cip, Genta, Ery and Pmb. LCA in combination with Genta had a synergistic bactericidal effect against *L. monocytogenes*, *S. aureus* and *S. suis*. Four other cholic acid derivatives, including chenodeoxycholic acid, ursodesoxycholic acid, hyodeoxycholic acid and cholic acid, were also conducted to evaluate the synergistic effect with Genta. Interestingly, chenodeoxycholic acid as a cholic acid derivative in our study also improved the bactericidal effect of Genta against Gram-positive bacteria. However, LCA treatment did not affect the growth of *L. monocytogenes* at this concentration. 

Thus, LCA treatment effectively improved the sensitivity of Gram-positive bacteria to Genta without affecting the viability of these pathogens. Bile acid can bind to the outer membranes and dissipate electrical potential to cause Gram-positive bacteria death [22]. This above effect may be related to the action of LCA against Gram-positive bacteria. Bile acids conjugated with β-lactam antibiotics showed excellent antifungal and antibacterial activity against *C. neoformans*, *S. aureus* and *C. albicans* [23]. 

In our study, although LCA or Genta had no visible destructive effect on *L. monocytogenes* membrane integrity, LCA in combination with Genta perturbed the bacterial membrane, which may increase the permeability of the membrane and facilitate the intake of Genta into cells [24]. Previous work has reported that the uptake of aminoglycosides may rely on bacterial respiration or proton motive force on the surface of the bacterial membrane [25], suggesting that the destruction of the *L. monocytogenes* membrane by LCA contributes to the observed synergistic bactericidal effect.

Furthermore, LCA treatment effectively improved the ability of Genta to eradicate *L. monocytogenes* biofilms. To further explore this mechanism, investigation into the synergistic effect between LCA and Genta on different bacterial species should be conducted, and the potential synergistic effect of LCA combined with other antibiotics against Gram-negative bacteria should be studied. Our results established that LCA improved the bactericidal effect and antibiofilm activity of Genta against *L. monocytogenes*. In addition, LCA in combination with Genta showed a synergistic bactericidal effect against Gram-positive bacteria, which could initiate the development of an anti-infectious agent to be used in combination with Genta for the treatment of infections caused by Gram-positive bacteria, including foodborne illnesses.

## 4. Materials and Methods

### 4.1. Microbial Strains, Reagents and Growth Conditions

The microbial strains used in this work are displayed in Table 1 and Table 2. LCA and its derivative (98.58% purity) were purchased from Shanghai Yuanye Biological Technology Co. Ltd. (Shanghai, China). The following antibiotics were obtained from Dalian Meilun Biotechnology Co. Ltd. (Dalian, China) or Beijing Dingguo Changsheng Biological Technology Co. Ltd. (Beijing, China), including Pmb, Amp, Genta, Tet, Ery, Cip, Lin and Cpl. Dimethyl sulfoxide (DMSO) was obtained from Sigma-Aldrich (St. Louis, MO, USA). 

Antibiotics were dissolved in sterile water at 5 mg/mL. LCA and its derivative were dissolved in DMSO at 40 mg/mL. The BacLight LIVE/DEAD bacterial viability kit was purchased from Invitrogen (Carlsbad, CA, USA). *L. monocytogenes* and *Staphylococcus aureus* (*S. aureus*) were cultured in tryptone soybean broth (TSB) [26,27], and *Streptococcus suis* type 2 (*S. suis*) was cultured in Todd Hewitt Broth (THB) supplemented with 2% yeast extract [28]. *Escherichia coli* ATCC25922 (*E. coli*), *Salmonella enterica serovar Typhimurium* SL1344 (*Salmonella* SL1344) and *Acinetobacter baumannii* ATCC19606 (*A. baumannii*) were cultured in Luria Bertani (LB) broth [29]. All the tested bacteria were inoculated from frozen at −80 °C into broth and incubated under aerobic conditions at 37 °C for 12 h.

### 4.2. MIC and FIC Index Determination

A checkerboard microdilution method was performed to determine the synergistic effect between the antibiotics and LCA against a variety of bacteria [30]. LCA and various antibiotics were diluted with culture medium in centrifuge tubes. Then, LCA and antibiotics were added to 96-well microtiter plates to obtain various combinations of LCA ranging from 0 to 256 μg/mL and different antibiotics. The bacteria were diluted to 5 × 10^5^ CFU/mL in all wells of 96-well microtiter plates. The fractional inhibitory concentration index (FIC index) was calculated according to the following formula after incubating statically at 37 °C for 16 h. The lowest concentration that inhibited bacterial growth was considered the MIC value of the individual and combined antibiotic.

$$\mathrm{FIC}\;\mathrm{index}=\frac{\mathrm{MIC}\;\mathrm{of}\;\mathrm{drug}\;A\;\mathrm{in}\;\mathrm{combinations}}{\mathrm{MIC}\;\mathrm{of}\;\mathrm{drug}\;A\;\mathrm{alone}}+\frac{\mathrm{MIC}\;\mathrm{of}\;\mathrm{drug}\;B\;\mathrm{in}\;\mathrm{combinations}}{\mathrm{MIC}\;\mathrm{of}\;\mathrm{drug}\;B\;\mathrm{alone}}$$

The FIC index was used to determine whether synergism, additivity, indifference or antagonism occurred between LCA and the antibiotics These interactions were defined as follows: synergism, FIC index ≤ 0.5; additivity, 0.5 < FIC index ≤ 2; indifference, 2 < FIC index ≤ 4; and antagonism, FIC index > 4.

### 4.3. Growth Curve Assay 

In order to determine the effect of LCA on the growth of *L. monocytogenes*, a growth curve assay was performed via plate counts [31]. Overnight cultures of *L. monocytogenes* were grown with shaking at 37 °C and were diluted (1:100) in TSB to an optical density (OD) at 600 nm of 0.3. LCA was added to bacterial cultures at different concentrations (0, 4, 8 and 16 μg/mL). Then, bacteria were cultured at 37 °C under aerobic conditions. The growth of bacteria was determined by the plate count method every 2 h for 8 h.

### 4.4. BacLight LIVE/DEAD Staining Experiments

LIVE/DEAD staining experiments were further carried out to determine the synergistic bactericidal effect of LCA and Genta against *L. monocytogenes* [32]. Overnight cultures of *L. monocytogenes* were diluted (1:50) in fresh culture medium containing 8 μg/mL LCA, 0.125 μg/mL Genta or combinations of LCA and Genta and incubated with shaking at 37 °C for 3 h. Following centrifugation (12,000 rpm for 1 min), the pellet was suspended in phosphate buffer solution (PBS) and adjusted to an OD 600 nm of 0.3. The samples were incubated with staining reagents at room temperature for 15 min in the dark and observed with a laser scanning confocal microscope (FV1000, Olympus, Tokyo, Japan) to examine the synergistic bactericidal effect of LCA and Genta against *L. monocytogenes*.

### 4.5. Scanning Electron Microscopy Analysis 

Scanning electron microscopy analysis was used to determine the bacterial membrane damage caused by combinations of LCA and Genta against *L. monocytogenes*. Overnight cultures of *L. monocytogenes* were diluted (1:50) in TSB containing 4 μg/mL LCA, 0.125 μg/mL Genta or combinations of LCA and Genta and then incubated with shaking at 37 °C. Then, *L. monocytogenes* was cultured statically in polylysine-coated slides in 24-well plates at 37 °C for 5 h until the absorbance value at 600 nm was 0.3. The slides were gently washed with PBS and treated with 2.5% glutaraldehyde at 4 °C for 12 h. Each sample was postfixed with 1% osmium tetroxide and dehydrated in ethanol. After vacuum freeze drying, the samples were coated with gold and visualized by a scanning electron microscope (Hitachi S3400, Tokyo, Japan).

### 4.6. Biofilm Inhibition Assays

Biofilm inhibition assays were conducted to measure the antibiofilm activity of combinations of LCA and Genta against *L. monocytogenes* [33,34]. Overnight cultures of *L. monocytogenes* were diluted in fresh culture medium to obtain a bacterial suspension at a concentration of 1 × 10^8^ CFU/mL.

Then, 1 mL of *L. monocytogenes* suspension was added to 24-well plates in triplicate and statically incubated at 37 °C for 24 h with different concentrations of LCA (4 μg/mL, 8 μg/mL and 16 μg/mL) combined with Genta (0.5 μg/mL). The unattached microorganisms were rinsed away with PBS three times. The biofilm of *L. monocytogenes* was stained with 0.1% crystal violet at room temperature for 15 min. After discarding the excess reagent from the 24-well plates, the plate was washed with phosphate buffer three times. The biofilm was dissolved in 95% ethanol prior to air drying at 55 °C for 10 min and quantified by determining the absorbance at OD 570 nm.

### 4.7. FIC Index Determination between Cholic Acid Derivative and Genta

In order to determine synergistic effect between other cholic acid derivatives (chenodeoxycholic acid, ursodesoxycholic acid, hyodeoxycholic acid and cholic acid) and Genta against the Gram-positive bacteria, a checkerboard microdilution method was performed. A various concentrations, cholic acid derivatives were mixed with different concentrations of Genta in 96-well microtiter plates. *L. monocytogenes*, *S. aureus* and *S. suis*. were diluted to 5 × 10^5^ CFU/mL in all wells of 96-well microtiter plates. After incubation at 37 °C for 14 h, FIC index was determined according to the above formulae.

### 4.8. Statistical Analysis

The FIC index was calculated according the formula in the MIC and FIC index determination section. Significant differences were analyzed with GraphPad Prism 5.0 using Student’s *t*-tests compared with no treatment as the control. All of our assays were performed three times. *p* values < 0.05 were considered significant as indicated in the figures.

## 5. Conclusions

Our results established that LCA improved the bactericidal effects and antibiofilm activity of Genta against *L. monocytogenes*. In addition, LCA in combination with Genta showed a synergistic bactericidal effect against Gram-positive bacteria, which could initiate the development of an anti-infectious agent to be used in combination with Genta for the treatment of infections caused by Gram-positive bacteria, including foodborne illnesses.

## Figures and Tables

**Table 1:** The MIC and FIC index determination of LCA combined with different antibiotics against Gram-positive bacteria.

Species	Sources	MIC (μg/mL)	Antibiotic	MIC (μg/mL)		FIC Index	Classification of the Interaction
		LCA Alone		Alone	Combination		
*S. aureus* USA 300	American Type Culture Collection		Ampicillin	128	8	0.18	synergism
			Erythromycin	32	4	0.25	
			Gentamicin	2	0.06	0.15	
			Ciprofloxacin	2	0.5	0.37	
		256	Lincomycin	1	0.5	0.62	
			Tetracycline	0.25	0.125	0.62	additivity
			Chloromycetin	4	2	0.62	
			Polymyxin B	128	16	0.25	synergism
*L. monocytogenes* EGD	Provided by Masao Mitsuyama		Ampicillin	0.5	0.5	1.25	additivity
			Erythromycin	0.125	0.125	1.25	
			Gentamicin	0.5	0.125	0.5	synergism
			Ciprofloxacin	0.	0.5	0.75	additivity
		32	Lincomycin	2	2	1.25	
			Tetracycline	0.25	0.25	1.25	
			Chloromycetin	4	4	1.25	
			Polymyxin B	32	8	0.5	synergism
*S. suis.* Type 2	An isolated strain from pig		Ampicillin	0.06	0.06	1.25	additivity
			Erythromycin	0.03	0.03	1.25	
			Gentamicin	16	4	0.5	synergism
			Ciprofloxacin	1	1	1.25	additivity
		16	Lincomycin	0.25	0.25	1.25	
			Tetracycline	32	32	1.25	
			Chloromycetin	1	1	1.25	
			Polymyxin B	8	8	1.25	

All assays were performed in triplicate. The concentration of LCA was 8 μg/mL in all bacterial isolates.

**Table 2:** The MIC and FIC index determination of LCA combined with different antibiotics against Gram-negative bacteria.

Species	Sources	MIC (μg/mL)	Antibiotic	MIC (μg/mL)		FIC Index	Classification of the Interaction
		LCA Alone		Alone	Combination		
*Salmonella* SL1344	Derived from the virulent strain SL1344		Ampicillin	2	2	1.01	additivity
			Erythromycin	256	256	1.01	
			Gentamicin	8	8	1.01	
			Ciprofloxacin	0.015	0.015	1.01	
		256	Lincomycin	1024	1024	1.01	
			Tetracycline	1	1	1.01	
			Chloromycetin	4	4	1.01	
			Polymyxin B	2	2	1.01	
*E. coli* ATCC25922	American Type Culture Collection		Ampicillin	1024	1024	1.01	additivity
			Erythromycin	128	128	1.01	
			Gentamicin	1024	1024	1.01	
			Ciprofloxacin	2	2	1.01	
		256	Lincomycin	1024	1024	1.01	
			Tetracycline	1	1	1.01	
			Chloromycetin	256	256	1.01	
			Polymyxin B	4	4	1.01	
*A. baumannii* ATCC19606	American Type Culture Collection		Ampicillin	1024	1024	1.01	additivity
			Erythromycin	8	8	1.01	
			Gentamicin	32	32	1.01	
			Ciprofloxacin	1	1	1.01	
		256	Lincomycin	1024	1024	1.01	
			Tetracycline	1	1	1.01	
			Chloromycetin	64	64	1.01	
			Polymyxin B	1	1	1.01	

All assays were performed in triplicate. The concentration of LCA was 8 μg/mL in all bacterial isolates.

**Table 3:** The MIC and FIC index determination of the cholic acid derivative combined with Genta against Gram-positive bacteria.

Species	Cholic Acid Derivative	MIC (μg/mL)	Antibiotic	MIC (μg/mL)		FIC Index	Classification of the Interaction
		Cholic Acid Derivative Alone		Alone	Combination		
*L. monocytogenes* EGD	chenodeoxycholic acid	256	Gentamicin	0.5	0.125	0.5	synergism
*S. suis.* Type 2		256		16	0.5	0.28	
*S. aureus* USA 300		256		2	0.03	0.26	
*L. monocytogenes* EGD	ursodeoxycholic acid	1024	Gentamicin	0.5	0.5	1.06	additivity
*S. suis.* Type 2		1024		16	8	0.56	
*S. aureus* USA 300		512		2	2	1.125	
*L. monocytogenes* EGD	cholic acid	2048	Gentamicin	0.5	0.5	1.03	additivity
*S. suis.* Type 2		512		16	16	1.12	
*S. aureus* USA 300		1024		2	1	0.56	
*L. monocytogenes* EGD	hyodeoxycholic acid	256	Gentamicin	0.5	0.5	1.25	additivity
*S. suis.* Type 2		256		16	8	0.75	
*S. aureus* USA 300		256		2	0.5	0.5	synergism

All assays were performed in triplicate. The concentration of the cholic acid derivative was 64 μg/mL for all bacterial isolates.

## Data Availability

The data that support the findings of this study are available from the corresponding author upon reasonable request.

## Sample Availability

Samples of the compounds that support the findings of this study are available from the corresponding author upon reasonable request.
